# The Role of the Microbiome in Food Allergy: A Review

**DOI:** 10.3390/children7060050

**Published:** 2020-05-26

**Authors:** Christina L. Nance, Roman Deniskin, Veronica C. Diaz, Misu Paul, Sara Anvari, Aikaterini Anagnostou

**Affiliations:** 1 Baylor College of Medicine, Section of Pediatric Immunology, Allergy and Retrovirology, Houston, TX 77030 USA; clnance@texaschildrens.org (C.L.N.); rxdenisk@texaschildrens.org (R.D.); Veronica.Diaz@bcm.edu (V.C.D.); misu.paul@bcm.edu (M.P.); sara.anvari@bcm.edu (S.A.); 2 Texas Children’s Hospital, Department of Pediatrics, Section of Immunology, Allergy and Retrovirology, Houston, TX 77030, USA

**Keywords:** allergic disease, microbiome, hygiene hypothesis, food allergy, asthma, atopy, atopic dermatitis, short chain fatty acids, T regulatory cells, dysbiosis, immune tolerance, G-protein coupled receptors, prebiotics, probiotics, fecal microbiota transplantation

## Abstract

Food allergies are common and estimated to affect 8% of children and 11% of adults in the United States. They pose a significant burden—physical, economic and social—to those affected. There is currently no available cure for food allergies. Emerging evidence suggests that the microbiome contributes to the development and manifestations of atopic disease. According to the hygiene hypothesis, children growing up with older siblings have a lower incidence of allergic disease compared with children from smaller families, due to their early exposure to microbes in the home. Research has also demonstrated that certain environmental exposures, such as a farming environment, during early life are associated with a diverse bacterial experience and reduced risk of allergic sensitization. Dysregulation in the homeostatic interaction between the host and the microbiome or gut dysbiosis appears to precede the development of food allergy, and the timing of such dysbiosis is critical. The microbiome affects food tolerance via the secretion of microbial metabolites (e.g., short chain fatty acids) and the expression of microbial cellular components. Understanding the biology of the microbiome and how it interacts with the host to maintain gut homeostasis is helpful in developing smarter therapeutic approaches. There are ongoing trials evaluating the benefits of probiotics and prebiotics, for the prevention and treatment of atopic diseases to correct the dysbiosis. However, the routine use of probiotics as an intervention for preventing allergic disease is not currently recommended. A new approach in microbial intervention is to attempt a more general modification of the gut microbiome, such as with fecal microbiota transplantation. Developing targeted bacterial therapies for food allergy may be promising for both the treatment and prevention of food allergy. Similarly, fecal microbiota transplantation is being explored as a potentially beneficial interventional approach. Overall, targeted bacterial therapies for food allergy may be promising for both the treatment and prevention of food allergy.

## 1. Introduction

Food allergies are estimated to affect approximately 8% of US children [1] and 11% of US adults [2]. Food allergy is defined as an adverse immune response to a food and can be either IgE-mediated (e.g., urticaria and anaphylaxis) or non-IgE mediated (e.g., food protein-induced enterocolitis syndrome and eosinophilic esophagitis) [3]. Food allergies pose a significant disease burden, which includes physical, dietary, social and psychological effects [4,5]. Currently, there is no cure for food allergies and the underlying cause has yet to be elucidated. IgE-mediated food allergy has been associated with both immune dysregulation and impaired gut epithelial integrity. In addition, there is increasing interest in a potential link between food allergy and the gut microbiome [6].

The microbiome consists of many microorganisms, including bacteria, fungi and viruses, as well as their genomic elements. There is increasing evidence that the human microbes residing in the airways, gastrointestinal tract and skin play an important role in normal health and disease states [7]. There are three different microbe–host relationships: (a) pathogenic, when the microbes harm the host; (b) commensal, when microbes coexist with the host without providing benefit or causing harm; and (c) symbiotic, when microbes and the host are mutually beneficial to each other [7].

Mucosal surfaces that line our gastrointestinal tract are continuously exposed to trillions of bacteria that form a symbiotic relationship and impact host health and disease. There are numerous studies evaluating the interactions between the host and microbiome including the dynamic changes in the commensal bacterial population, secretion, and absorption of metabolites. Imbalances in this relationship, or dysbiosis, contribute to the development of diseases such as inflammatory bowel disease, asthma, type 1 diabetes, cardiovascular disease, metabolic syndrome, obesity and allergic diseases such as asthma and food allergy [7,8].

In 1980, David Strachan coined the term “hygiene hypothesis” to explain the rise in atopic disorders. The hygiene hypothesis states that early childhood exposure to microorganisms protect against the development of allergic disease. A lack of exposure to these microbes due to reduced household sizes, improvements in household amenities, and higher standards of personal hygiene may have resulted in the impairment of immune tolerance and thus the rise in allergic disorders [9]. The hygiene hypothesis later evolved into the “old friends” hypothesis [10]. The “old friends” hypothesis provided a more detailed explanation between microbes and the development of inflammatory disorders. The “old friends” hypothesis proposed that microorganisms and their host have co-evolved symbiotically over thousands of years thereby allowing the organisms to play a key role in shaping the host immune response. A disruption in the symbiotic relationship could lead to immune dysregulation and promote allergic disease [10].

## 2. Methods

We performed a non-systematic review of published literature on “food allergy” and the “microbiome” from 2000–2020 using the PubMed database. We included clinical trials, meta-analyses, randomized controlled trials, reviews and systematic reviews in the English language. We identified key studies from the last 20 years, with special emphasis on the last decade to ensure we included recent research.

## 3. The Gut Microbiome

The gastrointestinal (GI) microbiome is a diverse community of bacteria, archaea, fungi, protozoa and viruses that colonize the mammalian GI tracts. The National Institute of Health (NIH) Human Microbiome Project identified more than 10^14^ microorganisms in the intestine. These microorganisms encompass >1000 species with essential physiological roles. The composition and concentration of these microbes differs along the tract and is affected by the host diet [11,12]. These bacteria have coevolved with humans over millennia and have a symbiotic relationship—humans consume prebiotic fiber, which is metabolized by resident microbes in the gut to create short chain fatty acids (SCFA). SCFAs, in turn, regulate immune responses [13]. The modern use of antimicrobial medications and the consumption of high-sugar, low-fiber diets have adversely affected the host–microbe interaction. There is emerging scientific evidence that the imbalance in the microbial flora (dysbiosis) contributes to altered host metabolism and predisposes humans to developing atopic diseases and an increased susceptibility to infections [14].

### 3.1. Host–Microbiome Interactions

Advances in technology have enhanced our understanding of the interplay between the gut microbiome and health and human disease. These include high-sensitivity means to study microbial communities in any type of ecosystem. Although the terms microbiota and microbiome are used interchangeably, microbiota are defined as the microbial taxa associated with complex organisms such as humans, whereas the microbiome is the catalog of these microbes and their genes [15]. Furthermore, the gut microbiota and their metabolic byproducts impact the host and regulate homeostasis by providing essential nutrients, affecting the health of the intestinal epithelial barrier, and regulating host innate and adaptive immune processes [16]. The disruption of a balanced microbiome can lead to immunological dysregulation with diseases such as inflammatory bowel disease (IBD), obesity and allergic diseases (including asthma and food allergy). An altered gut microbiome affects microbiota-derived products and metabolites, including pro- and anti-inflammatory materials [7,17]. Protection against microbial invasion is due to the intestinal barrier. This barrier has multiple lines of defense including commensal bacteria, which competitively inhibit the colonization of pathogenic bacteria and the production of metabolically protective compounds [18].

### 3.2. Early Life Microbiome

The timing of host–microbe interactions in early life appears to be important. The gut microbiome changes during the course of life, with the most rapid changes occurring in early life [19,20]. It is known that the overall diversity of the human gut microbiota increases steadily from birth until around 12 years of age, remains relatively stable throughout adulthood, and then declines in later years [21]. In adults, 60–70% of the gut microbiome is stable, with the degree of stability varying between phyla [21]. Additionally, the gut microbiome changes dramatically during pregnancy; intrinsic factors (e.g., stress) and extrinsic factors (e.g., diet, and drugs) influence the composition and activity of the gut microbiome throughout life [15,22].

## 4. The Microbiome and Atopic Diseases

There is growing evidence that the microbiome contributes to the development and manifestations of allergic disease. According to the hygiene hypothesis, mentioned previously, children growing up with older siblings have a lower incidence of allergic disease, compared with children from smaller families, likely due to their early exposure to environmental microbes in the home [9]. Additionally, factors associated with a decreased risk of developing allergic disease later in life include being born via vaginal delivery, being breastfed during the first 4 months of life, pet ownership, and the absence of early antibiotic exposure. By contrast, growing up in an urban, Westernized environment is associated with increased rates of asthma, atopic dermatitis and food allergy [7].

### 4.1. Effect of Environmental Exposures on the Composition of the Gut Microbiome and Atopic Diseases

Certain environmental exposures prenatally and during early life are associated with a diverse bacterial experience and reduced risk of allergic sensitization. The PASTURE study, a birth cohort study, showed an association between living in a farming environment with an increased environmental endotoxin exposure and a reduced incidence of asthma. The PARSIFAL and GABRIELA studies also demonstrated that there was a lower prevalence of asthma and other atopic diseases in children living on farms compared to suburban environments [23,24]. A diversity of microbial exposure was noted to be inversely associated with the risk of asthma in children growing up on farms in Central Europe and being exposed to a greater diversity of environmental fungi and bacteria [24]. Similarly, Wlasiuck et al. reported that the effect on asthma was largely due to exposure to cows, livestock, and straw, and the consumption of raw cow’s milk [25].

### 4.2. Infant Microbiome

The infant microbiome is shaped by race, diet, delivery method, and even the maternal microbiota of the gut and reproductive system during pregnancy [26,27]. The infant microbiome begins during fetal development with exposure to the uterine microbiome and meconium [19,26,28]. During fetal life, the fetal gut microbiota resembles the microbiota of amniotic fluid, which is colonized by bacteria. This includes organisms belonging to the *Enterobacteriaceae* family and bacteria from the *Firmicutes* phylum (such as *Lactobacillus species*, *Clostridium* species and *Bacillus* species) [19,26]. The method of delivery influences the infant’s microbiome as infants born by cesarean section (C-section) had microbiotas similar to the skin microbiota with *Staphylococcus, Corynebacterium* and *Propionibacterium* as the dominant species [19,29]. *Bacteroides* colonization is also delayed in infants born by C-section. Cesarean section has been associated with a higher risk of allergic rhinitis, asthma and autoimmune disorders such as celiac disease [19]. After birth, the microbiome is transiently dominated by *Enterobacteriaceae* and *Staphylococcus*. [19]. Savage et al. demonstrated that breastfeeding, and not formula feeding, was the dietary factor most consistently associated with the infant’s intestinal microbiome. Breastfed infants have higher numbers of *Bifidobacterium* species compared to formula fed infants. The latter group have higher *Clostridium* species and *Bacterioides* species [30]. As solid foods are introduced, bacteria from the *Firmicutes* phylum colonize the gut, and by about three years of age *Firmicutes* and *Bacteroides* are the main colonizers of the child gut microbiota.

Neonates have a limited capacity to initiate a Th1 response, and the fetus is generally very Th2-directed immunologically. Close immunologic interaction between the mother and fetus might lead to a Th2-skewed state noted in infants who develop allergic disease based on the evidence that non-allergic mothers have a lower Th2 response from mid to late gestation compared to mothers with atopy [28,31]. The bacterial colonization of the gut is also important in the differentiation of T helper (Th) cells into Th1, Th2, T regulatory cells (Tregs) and Th17 cells. Furthermore, intestinal bacteria such as *Lactobacillus, Bifidobacterium, Bacteroides* and *Clostridium*, as well as their metabolites, induce peripheral Treg cells that control inflammation in the gut and lungs. Th17 cells produce anti-inflammatory cytokines that improve barrier function and confer protection against pathogens.

### 4.3. Atopic Dermatitis and the Microbiome

Multiple studies have linked the microbiota of the respiratory tract, gastrointestinal tract and skin to allergic disease. The composition and diversity of the microbiome is different across body areas, with the GI tract showing the largest number and diversity of microbes [7]. Interestingly, the effects of microbial dysbiosis are not restricted to the local tissue environment. For example, the skin microbiota may affect the gut and gastrointestinal system [32]. Atopic dermatitis (AD) is characterized by an abnormal skin barrier, a result of multiple factors (e.g., genetic, environmental and immunologic). AD may predispose to the development of other atopic conditions, such as food allergy, allergic rhinitis and asthma (this process is known as the “atopic march”). Epithelial dysfunction linking the microbiome and immune dysregulation can lead to the atopic march. For example, disruption in the skin barrier allows increased allergen permeability and sensitization in addition to colonization by pathogenic organisms [32]. As a result of this, a Th2-type response is induced, causing further breakdown of the epithelial barrier. The Th2 response is generalized and affects distant sites such as the intestinal and respiratory tracts [32]. In AD, dysbiosis is characterized by reduced skin bacterial diversity, with increased *S. aureus* and decreased *S. epidermis*. Colonization and infection with *S. aureus* has been linked to increased IgE responses and severity of AD disease [7]. Commensal microbes, on the other hand, such as *S. epidermidis*, have the potential to inhibit *S. aureus* growth and improve the skin barrier by improving tight junctions and producing antimicrobial peptides [7]. Exposure to food allergens via a disrupted skin barrier has been shown to be a risk factor for food allergy [33]. Additionally, AD patients show increased intestinal permeability and a defective (“leaky”) gut barrier, enabling food allergen penetration and sensitization via the gut. A large-scale birth cohort study showed *E. coli* and *C. difficile* overgrowth in infants with AD; this was associated with a decreased number of beneficial bacteria, abnormal gut barrier function and loss of immune tolerance [34].

### 4.4. Asthma and the Microbiome

In asthma, living in an environment with diverse microbial flora has been shown to be protective against allergic inflammation and disease. Stein et al. examined two separate agricultural populations in the US, the Amish and the Hutterites. Both groups have similar lifestyles, but different farming practices—the Amish follow traditional practices, and the Hutterites use industrialized practices [35]. The investigators showed that the prevalence of asthma and allergic sensitization was four and six times lower in the Amish, respectively. Additionally, median endotoxin levels in Amish house dust were 6.8 times higher compared to those in the Hutterite houses. Differences in the immune response were also observed between the two populations—the genes most expressed in the Amish children were related to anti-inflammatory effects (limited activity of pathways depending on NF-kβ), whereas those expressed in the Hutterites were related to the activation of NF-kβ [35]. The authors concluded that differences in the Amish farming environment, with increased microbiome exposure, appeared to be protective against asthma through the modulation of the innate immune response [35].

## 5. The Gut Microbiome and Food Allergy

The gut microbiome has gained significant interest in recent years due to its many immuno-modulatory properties and role in mucosal tolerance. Gut dysbiosis likely precedes the development of food allergy, and the timing of such dysbiosis appears to be critical [36]. Multiple microbial orders have been implicated in food allergy, most commonly *Clostridiales* and *Lactobacillales* (beneficial effects), as well as *Bacteroidales* and *Enterobacteriales* (beneficial and detrimental effects have been described) [7]. Studies evaluating the profile of the gut microbiome have revealed unique microbial differences in patients with food allergies compared to healthy patients, and these studies have provided evidence that the dysbiosis of the microbiome precedes the development of food allergy [36]. Since food allergy is a complex and heterogenous disease and studies often involve very different patient populations and enrolment criteria, the results have not been consistent across various research projects. There has been, however, an increasing interest in investigating microbiome signatures related to food allergy.

### 5.1. Microbiome Signatures in Food Sensitization and Food Allergy

A study of 82 children with AD examined fecal microbiome signatures for food allergy. Sixty-two children presented with AD and food allergy, whereas 20 children had AD only (no food allergy). There were no differences in microbial diversity between the two groups. However, there were differences in microbial species; children with AD and food allergy had relatively more *E. coli* and *B. pseudocatenulatum* and less *B. breve, B. adolescentis, F. prausnitzii* and *A. muciniphila* than children with AD and no food allergy [37].

In infants, microbial colonization is complete within the first month of life; significant changes occur subsequently during weaning to solid foods. The first two years of life are crucial for the further development of the intestinal microbiota, with the first months of life considered to represent a critical window of interaction between the infant gut microbiota and the immune system [38]. Delayed colonization with commensal bacteria or alterations in the microbiota profile during infancy may predispose to the development of immune-mediated disorders such as allergic diseases [38]. Early colonization with potentially more pathogenic bacteria (e.g., *C. difficile* or *S. aureus*) has been linked to food allergy development, whereas colonization with more beneficial bacteria (e.g., *Bifidobacteria*) is seen more often in non-allergic children [38].

A US study collected and analyzed intestinal microbiome samples from infants at 3–6 months of age—the study examined 131 infants without food sensitization, 87 with food sensitization, 202 without food allergy and 14 infants with food allergy in this cohort [39] The investigators showed that the genera *Haemophilus, Dialister, Dorea* and *Clostridium* were underrepresented in infants who were sensitized to food, whereas the genera *Citrobacter, Oscillospira, Lactococcus* and *Dorea* were underrepresented in infants with food allergy. No differences were found in microbial diversity between the groups of sensitized and food-allergic versus the infant controls (non-sensitized and non-food allergic) [39].

A smaller study from Taiwan evaluated differences in the gut microbiota between 23 children with food sensitization and 22 healthy controls. The mean age of subjects was 14 months (range: 6–23 months). The investigators reported lower diversity of the total microbiota and the bacterial phylum *Bacteroidetes* in children with food sensitization. Additionally, the number of *Bacteroidetes* bacteria was significantly decreased and that of *Firmicutes* was significantly increased compared with those in the healthy children. At the genus level, there were significant increases in the numbers of *Sphingomonas, Sutterella, Bifidobacterium, Collinsella, Clostridium sensu stricto, Clostridium IV, Enterococcus, Lactobacillus, Roseburia, Faecalibacterium, Ruminococcus, Subdoligranulum* and *Akkermansia* in the food-sensitized children. The above group showed significant decreases in the numbers of *Bacteroides, Parabacteroides, Prevotella, Alistipes*, *Streptococcus* and *Veillonella* [40].

### 5.2. Microbiome Signatures in Egg and Cow’s Milk Allergies

In terms of investigating specific food allergies, a total of 141 children aged 3–16 months (median age: 9.5 months) were enrolled from five different centers in the United States with the aim of characterizing the gut microbiomes of egg-allergic subjects and controls [41]. In this study, 66 children had egg allergies and displayed enrichment of the *Lachnospiraceae* and *Streptococcaceae* families, while *Leuconostocaceae* were enriched in the healthy control group (non-food allergic children). Additionally, egg sensitization was associated with greater gut microbiome diversity and the *Lachnospiraceae* and *Ruminococcaceae* genera. Interestingly, neither compositional differences in the gut microbiome nor differences in gut bacterial diversity could predict the resolution or persistence of egg allergy at age 8 years. [41]. By contrast, a separate study investigating the association between early life gut microbiota composition and the resolution of cow’s milk allergy reported that enrichment of *Clostridia* and *Firmicutes* was noted in the infant gut microbiome (at 3–6 months of life) of subjects whose milk allergy resolved later in childhood [42].

Feehly et al. took this work a step further by colonizing germ-free mice with feces from either healthy infants or infants with cow’s milk allergy (CMA) [43]. The mice were subsequently sensitized with b-lactoglobulin (cow’s milk allergen) and the mucosal adjuvant cholera toxin. The investigators reported that mice colonized with feces from cow’s milk allergic infants were highly susceptible to anaphylaxis in response to a beta-lactoglobulin allergen challenge; a drop in core body temperature was noted as an indicator for anaphylaxis in the mice. All of the mice that received microbiota from the healthy infant donors were protected from an anaphylactic response to a beta-lactoglobulin challenge. Microbial analysis showed no difference in community diversity and evenness between the healthy and CMA-colonized mouse groups, but a *Clostridial* species, *Anaerostipes caccae*, was identified as protective against an allergic response to food. These findings underscore the importance of commensal bacteria in regulating responses to dietary antigens and potentially preventing allergic responses to food [43].

Interestingly, increased gut microbial diversity is suggested to be protective of allergic disease by some investigators, but not by others. This discrepancy may suggest that there is not always a beneficial association between increased microbiome diversity and atopic disease. Additionally, the role of the microbiome cannot be captured by a single dimension (e.g., alpha diversity), but involves more complex interactions between different taxa and their metabolic effects [41].

## 6. Nutrition and the Microbiome in Food Allergy

Nutrition is an important environmental factor in early life and may influence the immune system’s maturation and development in multiple ways. The first oral exposure to food allergens has the potential to mediate unresponsiveness and lead to oral tolerance, as seen in recent research studies on peanut allergy [44]. This observation has resulted in a significant change in infant feeding practices in the US and worldwide, with most Westernized countries now recommending allergenic food introduction at age 6 months and certainly within the first year of life [45].

### 6.1. Dietary Factors and the Microbiome

A variety of dietary factors appear to play a key role in the development of food allergy. Roduit et al. investigated the feeding practices during the first year of life of 856 children in a birth cohort study, while also prospectively collecting data on environmental factors and allergic disease development. They reported that an increased diversity of complementary food introduced in the first year of life was inversely associated with food allergy and food sensitization development up to 6 years of age. A dose–response effect was also noted with each additional food item introduced. Additionally, the investigators showed that increased food diversity was associated with an increased expression of forkhead box protein 3 (Foxp3), a marker for regulatory T cells (Tregs), suggesting a protective effect of a diverse diet against food allergy development. By contrast, children with a low food diversity score showed a reduced expression of Foxp3 [46].

### 6.2. The “Nutrition–Gut Microbiome–Physiology Axis”

Host diet and nutrition are key in maintaining a healthy microbiome. Mckenzie et al. describe the “nutrition–gut microbiome–physiology axis” as an essential link between diet, gut microbiota and allergic disease [47]. Short chain fatty acids (SCFAs) are metabolites produced by intestinal bacteria through the fermentation of non-digestible fibers. There is long-standing evidence that links high-fiber diets to better health outcomes [48]. By contrast, the Western diet is “pro-inflammatory”, due to its high fat, low fiber and highly processed nature, which appear to contribute to gut dysbiosis. SCFAs have been highlighted as key signaling molecules enabling “cross-talk” between the gut microbiome and the host, with emerging evidence of their importance in food allergy [48].

Roduit et al. analyzed SCFA levels in fecal samples from 301 children at the age of 1 year from a birth cohort. They reported that children with the highest levels of butyrate and propionate at the age of one year were less likely to suffer from asthma at the ages of 3 and 6 years. These children also showed significantly less allergic sensitization, and decreased risks of a food allergy and allergic rhinitis diagnosis. The authors suggest that increasing SCFA levels may be an option for preventing allergic disease in children [49]. More recently, Cait et al. examined whether bacterial butyrate production in the gut during early infancy is protective against the development of atopic disease in children [50]. The investigators found that the microbiome of infants who went on to develop allergic sensitization later in childhood lacked genes encoding key enzymes for both carbohydrate breakdown and butyrate production [50].

## 7. The Microbiome and Underlying Mechanisms in Food Allergy

The crosstalk between gut microbiota, host epithelial cells, and immune cells making up the gut-associated lymphoid tissue (GALT) is complex. Hence, having a tolerogenic phenotype depends on maintaining a balanced repertoire of intestinal microorganisms, a heathy gut epithelial barrier, and appropriate immune cell recruitment/activation. Imbalances in this host–microbe axis cause the breakdown of immune tolerance to food antigens that leads to allergic disease. In general, the microbiome affects food tolerance via the secretion of microbial metabolites and the expression of microbial cellular components (pathogen-associated molecular patterns, PAMP) [51].

### 7.1. SCFA Interaction with Host Epithelial and Immune Cells

Intestinal microorganisms such as *Faecalibacterium prausnitzii, Clostridium leptum* and *Eubacterium rectal* produce a large repertoire of metabolites including short-chain fatty acids (SCFA), aryl hydrocarbon receptor (AHR) ligands and polyamines [52]. The importance of SCFAs in immune tolerance has been extensively characterized at the genetic, biochemical and clinical levels. The major SCFAs are butyrate, propionate, acetate and valerate, which are metabolic byproducts of the microbial fermentation of SCFAs, present in varying concentrations throughout the gut but most abundant in the colon [52]. Total SCFA levels are also affected by the host genotype/ethnicity [53]. SCFAs directly engage G-protein coupled receptors (GPCR) on intestinal epithelial cells (e.g., GPR41, GPR43, GPR109A and Olfr78) or can passively diffuse through the cell membrane to inhibit histone deacetylases (HDAC) in epithelial and intestinal immune cells [53,54]. The downstream effect on enterocytes is regulating the expression of genes involved in energy metabolism, cell proliferation and differentiation, and fortifying the epithelial barrier (tight junctions and mucus production) [51]. SCFAs also effect gut inflammatory and tissue repair processes by altering NLRP3 inflammasome and autophagy activity [54,55].

SCFAs have direct immune-modulatory effects in the intestinal milieu and peripherally [52,56]. SCFAs induce gut dendritic cells (DC) to express retinal aldehyde dehydrogenase (RALDH), which produces retinoic acid from Vitamin A [57]. Secreted retinoic acid upregulates expression of the gut-homing integrins α4β7 on peripheral regulatory T cells (Treg). Treg cells constitute 20%–30% of the CD4 T cell population in intestinal lamina propria, and they promote immune tolerance by suppressing bystander T cells [56]. Separately, SCFAs regulate host antibody responses by altering metabolic pathways in B cells. SCFAs increase intracellular concentrations of acetyl-CoA and enhance oxidative phosphorylation, glycolysis and fatty acid synthesis [58]. The net effect is an increased production of IgA and IgM. Additionally, butyrate stimulates the production of protective cytokines (IFNγ and IL-10) in peripheral blood mononuclear cells (PBMCs) [58]. IL-10 induces the expansion of Treg cells and also suppresses T helper 17 (Th17) and Th2 cells, which are anti-pathogenic but pro-inflammatory cell types. Finally, in vitro studies show that SCFAs inhibit HDAC expression in human macrophages and PBMCs and downregulate the expression of inflammatory cytokines (IL-6, IL-8 and TNFα) [59,60]. The butyrate inhibition of HDAC3 promotes the differentiation of monocytes into macrophages and induces antimicrobial activity in vivo [61]. Overall, SCFAs are essential for maintaining immune homeostasis in the gut by regulating protective and inflammatory responses.

In vivo and clinical studies have validated the importance of SCFAs in food allergy. For example, Vonk et al. demonstrated that butyrate supplementation enhances oral immunotherapy (OIT) desensitization in a murine model of cow’s milk allergy (CMA) [62]. In humans, in studies with pediatric patients with CMA, the ingestion of hydrolyzed formula containing a butyrate-producing probiotic *L. rhamnosus* GG (LGG) increased fecal butyrate levels and was associated with the acquisition of immune tolerance [63]. Furthermore, compared to healthy children, children with atopic disease have lower levels of fecal butyrate and valeric acid [64]. Children who developed tolerance to peanut and egg had an increased frequency of IL-10-expressing Tregs with concomitant higher blood levels of IL-10 [65]. Consistent with the in vitro findings is the observation that butyrate supplementation increases the number of activated (FoxP3+)Treg cells. In children, developing tolerance to CMA correlates with a higher frequency of Treg cells and a reduced proliferation of milk-specific T cells [66]. By contrast, germ-free mice and antibiotic-treated mice who develop allergic disease have lower numbers of colonic Treg cells [67]. Similarly, patients with dysregulation in FoxP3+ Treg (Immune dysregulation, polyendocrinopathy, X-linked; IPEX) develop food allergies among other GI and immune pathologies [68].

### 7.2. Polyamines, AHR Ligands and Gut Homeostasis

Polyamines (PA) are very reactive polycationic molecules mostly derived from the diet (e.g., soybeans, mushrooms, beef and pork). PAs are also produced de novo by commensal bacteria (*Bacteroides* species) and host cells [69]. Spermidine, spermine, putrescine and cadaverine are the predominant PAs produced by gut bacteria. Polyamines are essential for maintaining the gut epithelial barrier function (upregulating junctional proteins, e.g., occludins and E-cadherins) in in vitro studies [70]. The role of polyamines in preventing food allergies is limited but an active topic of investigation [71,72,73].

Aryl hydrocarbon receptors (AHR) are ubiquitously expressed transcription factors that sense xenobiotic ligands. AHR ligands include tryptophan metabolites derived from microbiota and dietary macronutrients. The activation of the AHR signaling pathway is important for maintaining gut health including a direct effect on mucosal immunity [74]. For example, intestinal AHR signaling is important for intestinal crypt stem cell differentiation [75]. Additionally, exogenous AHR ligands activate intestinal intraepithelial lymphocytes and affect epithelial barrier function [76]. Furthermore, in animal studies, mice fed a diet deficient in AHR ligands had lower fecal IgA compared to mice consuming a regular chow diet [77]. In murine studies on peanut allergy, activating AHR pathways decreases peanut sensitization by shifting the naïve T cell population to a Treg phenotype [78,79]. To our knowledge, there are currently no clinical studies evaluating the functional effect of AHR ligands on food tolerance.

## 8. Microbiome and Food Allergy Treatments in the Horizon

### 8.1. Prebiotics and Probiotics in Food Allergy

With the evidence to support that the gut microbiome plays a role in the development of food allergies, the investigation and understanding of microbiome therapy has been of interest in providing a potential avenue for food allergy prevention and treatment.

Microbiome therapy entails the use of both prebiotics and probiotics. The term prebiotics was first defined in 1995, and since that time, the definition of prebiotics has evolved. Currently, prebiotics are described as a “substrate” that is selectively utilized by host microorganisms, leading to specific changes in the composition and/or activity of the gastrointestinal microbiota, thereby providing beneficial health effects [80]. Additionally, prebiotics include non-digestible compounds, such as oligosaccharides or soluble fermentable fibers that are selectively utilized and promote the growth of beneficial microorganisms and improve health. Prebiotics pass through the upper gastrointestinal tract undigested and serve to stimulate the activity and/or growth of microorganisms colonized in the large intestines [80]. By contrast, probiotics are live microorganisms that provide beneficial health benefits to the host. When given in adequate doses, probiotics may provide health benefits through the modulation of immune responses. When paired together, products containing both prebiotics and probiotics are known as symbiotics. A few studies have provided evidence that the composition of the gut microbiota can influence the development and course of food allergy. Therefore, the use of prebiotics, probiotics or synbiotics has been investigated for their potential benefits on the host, in order to prevent and/or treat food allergies.

#### 8.1.1. Prebiotics

The consumption of prebiotics has been suggested to provide more favorable microbial colonization patterns and potentially allow for the development of tolerance and the prevention of allergy. There are limited studies investigating the role of prebiotic supplementation on food allergy prevention. A systematic review in 2013 examined whether prebiotics given to infants could prevent infant sensitization to dietary allergens [81]. The authors of the systematic review concluded that prebiotic supplementation could potentially prevent eczema in infants up to two years of age; however, neonatal prebiotic supplementation did not prevent the development of food allergies [81]. Overall, more research is needed to evaluate the role of prebiotics in the development of allergic disease, including food allergies.

#### 8.1.2. Role of Probiotics in Preventing Food Sensitization

With regard to probiotics, few clinical trials have investigated whether the use of probiotics has a beneficial effect on preventing the development of atopy and food sensitization. In 2015, a systematic review by Cuello-Garcia et al. evaluated 29 randomized trials, with 15 trials (*n* = 3509) evaluating probiotic use during pregnancy, 13 trials (*n* = 1595) evaluating the use of probiotics in breastfeeding mothers and 15 trials (*n* = 3447) evaluating the use of probiotics in infants. The authors concluded that probiotics reduced the risk of eczema when taken during the last trimester of pregnancy, during breastfeeding or during infancy; however, the evidence was not strongly supported. Additionally, the prevention of food allergies and other allergic conditions was not observed with probiotic use during pregnancy, breastfeeding and/or when given to infants [82]. The World Allergy Organization (WAO) also conducted a systematic review of randomized controlled trials of probiotics for allergy prevention. The WAO concluded that there was weak evidence to support that probiotic supplementation could reduce the risk of developing allergic disorders in pediatric patients; however, a small risk reduction for eczema prevention could be observed with probiotic use [82]. A 2016 meta-analysis examined 17 trials in order to determine whether prenatal and postnatal probiotic administration could reduce the risk of atopy and food sensitization. The authors of the meta-analysis examined 17 trials and concluded that with a combined approach of maternal supplementation during pregnancy and infant supplementation of probiotics, a beneficial effect was observed in preventing the development of atopy and reducing the risk of food sensitization (RR 0.77, 95% CI: 0.61–0.98); however, the protective effect was not observed when probiotics were not given during both periods [83].

#### 8.1.3. Role of Probiotics in Accelerating Tolerance to Foods

There have also been studies investigating the use of probiotics in patients with a confirmed food allergy diagnosis. In 2008, a double-blind randomized, placebo-controlled trial evaluating 119 infants with food challenge-confirmed cow’s milk allergies examined whether extensively a hydrolyzed formula supplemented with both probiotics *Lactobacillus casei* and *Bifidobacterium lactis* for 12 months could accelerate tolerance to cow’s milk in infants with cow’s milk allergy. No difference was observed in the development of cow’s milk tolerance between the treatment (77%) and placebo groups (81%) [84]. By contrast, a subsequent study examined 55 infants with food challenge-confirmed cow’s milk allergies who received either extensively hydrolyzed casein formula (EHCF) or EHCF supplemented with *Lactobacillus* GG (LGG). EHCF supplemented with LGG augmented the development of tolerance to cow’s milk protein after 6 months and 12 months of treatment compared to that in the control group [85]. Additionally, the same investigators examined the effects of the supplementation of EHCF with/without LGG for 3 years in 220 children with IgE-mediated cow’s milk allergy. The authors demonstrated that the infants with IgE-mediated cow’s milk allergy receiving the EHCF supplemented with LGG had developed cow’s milk tolerance over the course of the 3-year treatment period. The incidence of developing other atopic conditions was reduced in patients receiving EHCF with LGG [86].

#### 8.1.4. Probiotics in Peanut Allergy

Probiotics have also been examined in patients with peanut allergy undergoing peanut oral immunotherapy. In 2015, Tang et al. reported the first results of the effects of probiotics in patients receiving peanut oral immunotherapy (POIT). The study compared 31 peanut allergic children receiving daily POIT with the probiotic *Lactobacillus rhamnosus* CGMCC 1.3724 and 31 peanut allergic children receiving daily POIT with placebo (maltodextrin) for a total of 18 months. The study’s primary outcome was to evaluate sustained unresponsiveness 2 to 5 weeks following the discontinuation of POIT treatment. The authors reported that 82.1% of the probiotic and POIT group achieved sustained unresponsiveness compared to 3.6% of the placebo and POIT group (*p* < 0.001). Additionally, 89.7% of those receiving the probiotic and POIT were desensitized compared to 7.1% of those receiving the placebo (*p* < 0.001) [87]. The follow-up study, 4 years following treatment cessation, reported that 67% of the subjects who received the probiotic and POIT treatment were consuming peanuts compared to 4% of the patients who received the placebo and POIT. Additionally, the subjects who received the probiotic and POIT had smaller peanut skin prick wheal sizes and significantly higher peanut sIgG4/sIgE ratios compared to subjects who received the placebo and POIT [88]. Although both studies suggested that probiotics could help patients achieve peanut tolerance, the research was limited by the absence of a probiotic-only or POIT-only control group. The evidence at this time for probiotics, as either a preventative or therapeutic agent for food allergies, remains low, and probiotics are currently not recommended for routine use in food allergy prevention or treatment.

#### 8.1.5. Ongoing Studies on Synbiotics

Currently, an ongoing clinical trial (TEMPO) investigating partially hydrolyzed infant formula with and without the added synbiotics *Bifidobacterium breve* M-16V plus the prebiotics oligofructose, long-chain inulin and acidic oligosaccharides is being examined in 851 infants at risk of developing allergy. The primary outcome of the study will be to measure the levels of *Bifidobacterium breve* in stools at 17 weeks of age, and the secondary outcome will be to assess IgE-mediated allergic manifestations in the blood up to 52 weeks of age (*clinicaltrials.gov/ct2/show/NCT03067714*). In addition to the TEMPO study, a similarly designed study, known as the MAESTRO study, is investigating the effect of a partially hydrolyzed infant formula with added synbiotics on the development of allergic manifestations in infants at high risk of developing allergy up to the age of 12 months (*clinicaltrials.gov/ct2/show/NCT03062995*). Finally, the ongoing double-blind prospective trial (PRESTO) has been evaluating the development of tolerance to cow’s milk at 12, 24 and 36 months, in infants with IgE-mediated cow’s milk allergy, following 12 months of dietary management with an amino acid formula (AAF) supplemented with and without synbiotics (short and long chain fructo-oligosaccharides and *Bifidobacterium breve* M-16 V). (*trialregister.nl/trial/3567*).

### 8.2. Metagenomics and Metabolomics

Novel methodologies and approaches are now being implemented in the field of the microbiome as it relates to food allergy. These involve the analysis of diseased versus healthy microbial populations by metagenomics, metabolomics or other techniques to uncover key commensal microbial factors that influence disease pathology, gut homeostasis and the immune response [16]. Limited information was garnered in initial studies in this field. This includes harvesting and expanding microbiotia using culture-based approaches. These experiments provided limited information as the vast majority of microbiota cannot be grown in culture media. More recent studies have used next-generation sequencing, including 16S rRNA sequencing and metagenomic sequencing. The metagenome is the collective assembly of genomes from an environment (e.g., the gut) [89]. This enables a more comprehensive and culture-free profiling of taxa from collected specimens [20,25]. Shotgun metagenomic sequencing, in which the total DNA of an ecosystem is sequenced, provides a broad and deep characterization of all types of microbiota. It provides coverage and resolution of the lowest taxonomic levels (e.g., species) and the potential for the functional annotation of genes and proteins [36].

Metabolomics represents one of the meta-omic approaches to study gut microbiota functions. Metabolomics is the collective array of metabolites present in a biological sample [89]. Untargeted metabolomic profiling has emerged as a powerful technique to dissect altered pathways contributing to complex diseases. Metabolomics uses high throughput techniques to characterize and quantify small molecules in several biofluids, such as feces, urine, plasma, serum and saliva. The use of metabolomics is considered a powerful top-down systems biology approach, and it is essential to reveal the genetic–environment–health relationship, as well as the clinical biomarkers of diseases such as atopic disorders [89]. Ultimately, these types of studies can identify beneficial bacteria related to a targeted disease based on their metabolomic “fingerprint”. These specific bacteria can be cultured and then introduced into the patient to treat disease.

### 8.3. Fecal Microbiota Transplantation (FMT)

A new approach in microbial intervention is to attempt a more general modification of the gut microbiome using fecal microbiota transplantation (FMT). FMT is a type of bacteriotherapy (the purposeful use of bacteria or their products to treat illness), where stool (consisting of diverse microbe populations) from a healthy donor is transferred into the GI tract of a diseased individual with the goal of treating a particular disease. FMT may be performed by different routes, including colonoscopy, a nasogastric tube, or oral capsules [20,90]. Pathogenic or opportunistic microbes are out-competed by the newly introduced bacteria to re-shape the gut microbiome to benefit the host [16]. Compared to probiotic treatments that contain a few bacterial species, FMT involves thousands of bacterial species native to the GI. FMT increases microbial diversity, restoring the gut microbiota community structure and diversity to the level of a healthy person [16]. In humans, FMT for gut diseases has been well-studied for *Clostridium difficile* infection (CDI) [91,92] and has also been applied to antibiotic-resistant gastrointestinal infections [91]. FMT has been successfully used to treat patients with *Clostridium difficile* colitis with communities from healthy donors transferred to those with the condition [20,90].

In ground-breaking studies in murine models, the identification of specific microbiota was elucidated to launch FMT clinical trials in humans with food allergies. Studies showed that FMT using stools taken from healthy human infants and transplanted into a food-allergic mouse model protected them from anaphylaxis after allergen exposure, whereas anaphylaxis was not abrogated when FMT was performed with stools taken from food-allergic infants [90]. Other mouse studies, investigating clusters impacted by dysbiosis in infants, utilized a type of mouse, Il4raF709, that is genetically prone to food allergy [43,90,93]. Using FMT bacteriotherapy containing commensal Clostridiales strains with or without five Bacteroidales strains both prevented and treated food allergy. In addition, bacteriotherapy led to the upregulation of RoR gamma Treg cells. There was also a decrease in total and ova-specific serum IgE, IL-4 production and GATA3 expression by Treg cells. Protection from anaphylaxis was also noted when treating these mice with a single *Clostridial* species, *Subdoligranulum variabilis*, which was also impacted by the dysbiosis in food-allergic infants [90,93]. In a different food allergy mouse model, another species of *Clostridia* known as *Anaerostipes caccae* was protective against allergic reaction [43].

The research advances in the microbiome field have moved food allergy therapy into the arena of microbiome-related human clinical trials. FMT is an intriguing gut microbiome manipulation strategy to consider given that findings from murine models support that fecal transfer is an effective mode of altering allergic outcomes [20]. A small Phase I open-label trial to evaluate the safety and tolerability of oral encapsulated FMT administered in an open-label manner over 2 days for the treatment of peanut allergy in 10 adult subjects (18–40 years) is underway (NIH ClinicalTrials.gov #NCT02960074). The agent being tested is a screened-donor inoculum of frozen fecal material [20,90]. A study of the next-generation microbial transfer of rationally selected strains based on preclinical findings by Atarashi et al. is in a Phase I clinical trial for the treatment of peanut allergy (NIH ClinicalTrials.gov #NCT03936998). VE416 is a combination of dormant bacteria given as a capsule to subjects pretreated with vancomycin to facilitate the colonization of the transferred bacteria. In this ongoing study, VE416 or placebo will be given with peanut oral immunotherapy [20].

## 9. Conclusions

In conclusion, the microbiome plays an important role in early immunological development, and it is stipulated that early infancy may represent a key window for interventions that aim to manipulate the microbiome for the benefit of the human host. Microbial colonization begins shortly after birth, and multiple factors contribute to the development of normal flora. These factors include genetics, maternal–fetal interactions, the place and mode of delivery, infant feeding methods and the use of antibiotics. A negative family history of atopy, vaginal delivery, home birth, colostrum, breast milk (as a source of commensal bacteria) and the avoidance of antibiotic use in early life are all considered protective against food sensitization and allergy. By contrast, a positive family atopic history, caesarean delivery, hospital delivery, bottle-feeding and the use of antibiotics early in life are considered risk factors for atopic disease and food allergy development. Certain environmental exposures, such as growing up in a farming environment, have also been shown to protect from the development of atopic disease.

Additionally, there is both direct and indirect scientific evidence for the role of the microbiome and their metabolic byproducts such as short chain fatty acids in food allergy development. This underscores the important role of the microbiota in shaping the intestinal immune phenotype and provides invaluable insight into developing novel treatment strategies to restore symbiosis. Studies that have directly measured microbial diversity and composition in populations with and without food allergy have provided direct evidence that gut microbiota differ in individuals with food allergy. Advances in immunotherapy have highlighted the immunologic influence of commensal microbiota on oral tolerance in recent years. As a result, there is potential for the application of microbiota and/or microbial-derived products in food allergy treatment.

The routine use of probiotics as an intervention for preventing allergic disease, with the exception of eczema in high-risk infants, is not currently recommended. There are ongoing trials evaluating the benefits of probiotics and prebiotics, in addition to the optimal strains and dosages needed, as well as the duration of probiotic administration and how they may contribute to the prevention or treatment of atopic diseases.

Developing targeted bacterial therapies for food allergy may be promising for the treatment and prevention of food allergy. Understanding the biology of the microbiome and how it interacts with the host to maintain gut homeostasis will be key to developing smarter therapeutic approaches.

## Abbreviations

AHR	aryl hydrocarbon receptor
DBPC	double-blind placebo controlled peanut challenge
DC	dendritic cells
RALDH	retinal aldehyde dehydrogenase
EHCF	extensively hydrolyzed casein formula
FMT	fecal microbiota transplantation
GPCR	G-protein coupled receptors
HDAC	histone deacetylases
LGG	Lactobacillus GG
PBMCs	peripheral blood mononuclear cells
PA	polyamines
POIT	peanut oral immunotherapy
SCFAs	short chain fatty acids
T regs	T regulatory cells
Th1	T helper 1
Th2	T helper 2
Th17	T helper 17
CMA	cow’s milk allergy
